# 

**Published:** 1902-11

**Authors:** 


### Society Notes.

### The South Texas Medical Association.

The South Texas Medical Association will meet in Houston, December 16th and 17th (prox.).

D. S. WEIR, M. D., Secretary

### The Brazos Valley Medical Society—Announcement.

The fourteenth semi-annual meeting of the Brazos Valley Medical Association will convene at Rockdale, Texas, on the second Tuesday and Wednesday of November, 1902.

This meeting will certainly be one of the best ever held by the Association and all who will attend may be assured of a right royal welcome. The good people of Rockdale know how to carry this out to perfection. A review of this program will show that we have papers on obstetrical, surgical, medical and other subjects by the very brightest minds in our grand State. This alone will insure success, but we want every member to be present to assist. Don't fail to come.

To those who have written articles we would say: If you find it impossible to attend, be sure to send your papers to the secretary by the 10th inst. that they may be read and discussed before the Association.

F. R. COLLARD, President.

W. B. BRIGGS, Secretary.

---

*To the Members of the Brazos Valley Medical Association, their Wives, Daughters and Sweethearts:*

You are cordially invited by the physicians and citizens of Rockdale to attend the meeting of the Brazos Valley Medical Association at this place November 11 and 12, 1902.

Reception at the home of Dr. R. S. Wallis Tuesday from 8 to 12 p. m.

Reception and banquet by citizens Wednesday night.

R. S. V. P.

On account of the length of the program, a most interesting one, we are compelled to omit it. Copies of it will be distributed at the meeting.—ED.

